# Secondhand Smoke Exposure in Lao People’s Democratic Republic: Results From the 2015 National Adult Tobacco Survey

**DOI:** 10.3389/ijph.2021.1604436

**Published:** 2021-12-31

**Authors:** Shweta Vishwas Kulkarni, Phonepadith Xangsayarath, Daovieng Douangvichith, Latsamy Siengsounthone, Khatthanaphone Phandouangsy, Ly Thi-Hai Tran, Phuc Hong Le, Thanh Cong Bui

**Affiliations:** ^1^ Department of Biostatistics and Epidemiology, Hudson College of Public Health, University of Oklahoma Health Sciences Center, Oklahoma City, OK, United States; ^2^ National Center for Laboratory and Epidemiology, Ministry of Health of Lao PDR, Vientiane, Laos; ^3^ Ministry of Health of Lao PDR, Vientiane, Laos; ^4^ Lao Tropical and Public Health Institute, Ministry of Health of Lao PDR, Vientiane, Laos; ^5^ Secretary of the National Tobacco Control Taskforce, Department of Hygiene and Health Promotion, Ministry of Health of Lao PDR, Vientiane, Laos; ^6^ Integris Health, Oklahoma City, OK, United States; ^7^ Center for Value-Based Care Research, Cleveland Clinic Community Care, Cleveland Clinic, Cleveland, OH, United States; ^8^ Department of Family and Preventive Medicine, College of Medicine, University of Oklahoma Health Sciences Center, Oklahoma City, OK, United States; ^9^ TSET Health Promotion Research Center, Stephenson Cancer Center, University of Oklahoma Health Sciences Center, Oklahoma City, OK, United States

**Keywords:** tobacco control, global health, Lao PDR, smoke-free environments, tobacco use, secondhand smoke

## Abstract

**Objectives:** Second-hand smoke (SHS) exposure causes >600,000 deaths annually worldwide, however, information regarding SHS exposure in Lao People’s Democratic Republic (Lao PRD) is limited; we report SHS exposure prevalence at home, inside workplaces, and indoor public spaces in Lao PDR.

**Methods:** Data were from the 2015 Lao National Adult Tobacco Survey, a nationally representative sample of 7,562 participants aged ≥15 years recruited through a stratified 2-stage cluster sampling approach.

**Results:** 88.3% (83.9% of non-smokers) reported SHS exposure at home and 63.0% (54.0% of non-smokers) at workplaces. Among non-smokers, women had greater exposure at home than men (86.6 vs. 77.0%). Lower education levels were associated with exposure at home or the workplace. 99.2% reported SHS exposure at any public place; specifically for restaurants/food stores 57.7%, government offices 56.2%, public transport 31.6%, and health care facilities 11.7%.

**Conclusion:** SHS exposure at home and workplace in Lao PDR is among the highest in South-East Asia. Comprehensive smoke-free policies at government-owned workplaces and facilities, stricter enforcement of these smoke-free policies, and strategies to encourage smoke-free environments at homes and in public places are urgently needed.

## Introduction

According to the World Health Organization (WHO) report on the worldwide burden of disease from exposure to second-hand smoke (SHS), more than 600,000 annual deaths worldwide are caused by SHS [1]. Among non-smokers, lung cancer risk increases with both the duration and the level of SHS exposure [2]. Tobacco-related diseases account for 10.9 million disability-adjusted life years (DALYs) worldwide. Of all deaths attributable to SHS, 28% occur in children, and 47% in women [3]; SHS exposure can cause lung cancer [4], ischemic heart disease [5], asthma [6], and chronic obstructive pulmonary disease (COPD) among adults [7]. SHS exposure in pregnant women can result in low birth weights, while SHS in childhood can cause chronic respiratory symptoms, lower respiratory illness, asthma, middle-ear infection, reduced pulmonary function, and sudden infant death syndrome [1]. The WHO Framework Convention on Tobacco Control (FCTC) suggests creating smoke-free environments in all indoor workplaces, public places, and on public transport, while also instituting educational strategies such as voluntary smoke-free home policies to prevent tobacco-related mortality and morbidity [8].

In Lao People’s Democratic Republic (Lao PDR), cigarette smoking is a major public health problem; 50.8% of men and 7.1% of women smoke tobacco [9, 10]. The prevalence of tobacco use in Lao PDR is among the highest in the Southeast Asian region, with chronic exposure to tobacco smoke and impaired lung function being particularly high among men. The Lao government has implemented several of the tobacco control measures described in the WHO FCTC [11]. The National Assembly approved the National Tobacco Control Law in 2009 and the National Tobacco Control Committee was established in 2012. Some governmental institutions, including the Lao Women’s Union and the Ministry of Education, maintain 100% smoke-free facilities, while several other ministries are in the process of enforcing the smoke-free environment policy [12]. Warnings about the adverse health consequences of smoking are required on cigarette packaging. These tobacco control policies, together with nationwide media-based education campaigns to increase public awareness of the dangers of smoking, are associated with cessation attempts or intention to quit among smokers [10]. However, some other measures necessary for tobacco control, such as tobacco treatment programs/quitline or nicotine replacement therapy, are still not utilized in Lao PDR [11].

There is very limited information regarding the national prevalence of SHS exposure in Lao PRD. This study aims to examine the national prevalence of SHS exposure at home, inside the workplace, and indoor public places (e.g., government offices, health care facilities, restaurants/food stores, and public transport vehicles). The study findings will provide a basis for appropriate future policies and strategies to reduce SHS exposure and the associated morbidity and mortality burden in Lao PDR.

## Methods

Data were from the National Adult Tobacco Survey (NATS) conducted in 2015 in Lao PDR [9]. The Lao NATS consisted of a nationally representative sample of 7,562 participants ≥15 years old recruited nationwide through a stratified 2-stage cluster sampling approach [10]. Provinces served as strata (n = 18), and villages or comparable administrative units served as primary sampling units (PSU) in each stratum. The probability proportional to size method was used to select PSUs. At the second stage, 20 households were selected from each PSU through a circular systematic sampling method (total sample: 2,969 households). All the eligible people ≥15 years old in selected households were invited to participate. Almost all (99%) of the pre-identified households were contacted successfully (i.e., having correct addresses and occupied), had at least one eligible individual, and agreed to participate. The individual participation rate was 85%, with an average of 2.5 persons/household participating. The field data collection team included 4 supervisors and 12 interviewers. Data collection started in November 2014 and lasted for approximately 1 year. The survey included questions about demographics, tobacco uses practices, and exposure to SHS at different places. The questionnaire was administered by the CommCare software on tablets. CommCare is an open-source mobile platform used for data collection. It includes CommCareHQ (Web) and CommCare Mobile components [13, 14]. Comprehensive training was provided to all interviewers to ensure standardization.

The main dependent variables in this analysis were SHS exposure at home, SHS exposure in workplaces, and SHS exposure in public places. SHS exposure at home was considered present if the participant’s response to the question about having seen someone smoking inside the home was either “daily,” “weekly” or “monthly,” and was considered absent if the participant’s response was “less than monthly” or “no.” SHS exposure in workplaces and public places was assessed using questions asking the participants if they saw anyone smoking in each specific place during the past 30 days. Sociodemographic variables of interest include age, sex, residence, ethnicity, religion, marital status, educational attainment, occupation, and average income per household member per day in US dollars.

We used SAS 9.4 to perform statistical analyses and accounted for the complex sampling design and sampling weights (using PROC SURVEYFREQ). We first performed basic descriptive statistics to examine the variables. We used a generalized linear mixed model with a logit function to examine associations between dependent variables and independent variables of interest. We adjusted models for age groups, sex, residence, ethnicity, religion, marital status, average household income, and educational level for evaluating the association with SHS exposure at home with additional adjustment for occupation for evaluating association with SHS exposure at workplaces. All *p*-values were two-tailed and the results for the statistical tests were considered significant if *p* < 0.05.

## Results

The NATS results showed that 88.3% of Lao people (83.9% among non-smokers) reported SHS exposure at home (Table 1) and 63.0% (54.0% among non-smokers) reported SHS exposure at the workplace (Table 2). Participant characteristics have been reported in Supplementary Table S1. Among those who reported SHS exposure at home in the past 30 days, 71.5% were exposed to daily SHS (Supplementary Table S2) and 17.8% were exposed to weekly SHS. For all participants, the prevalence of SHS exposure at home was highest among the 45–64 years of age group (89.4%), while for non-smokers it was the highest in the 15–24 years of age group (86.7%). Considering non-smokers, women were more likely than men to report SHS exposure at home (86.6 vs. 77.0%) (Table 1). SHS exposure at the workplace was highest for Lao people ≥45 years of age (70.7% among all participants and 59.5% among non-smokers, Table 2).

**TABLE 1:** Prevalence of secondhand smoke exposure at home, Lao People’s Democratic Republic, 2015.

Characteristics	All participants (smokers and non-smokers)						Non-smokers					
	Unweighted count (N)	Weighted %^a^	Unadjusted prevalence ratio (95% CI)	*p-*Value	Adjusted prevalence ratio^b^ (95% CI)	*p-*Value	Unweighted count (N)	Weighted %^a^	Unadjusted prevalence ratio (95% CI)	*p-*Value	Adjusted prevalence ratio^b^ (95% CI)	*p-*Value
Total (N = 6,602)	5,837	88.3					3,601	83.9				
Age group (years)												
15–24	1,033	88.1	1.03 (0.89–1.19)	0.624	1.05 (0.97–1.14)	0.206	873	86.7	1.12 (1.04–1.21)	0.002	1.12 (1.10–1.45)	0.0007
25–44	2,485	87.7	1.02 (0.93–1.12)	0.772	1.00 (0.95–1.07)	0.765	1,600	83.4	1.08 (0.99–1.17)	0.057	1.21 (1.07–1.38)	0.002
45–64	1896	89.4	1.01 (0.96–1.06)	0.298	1.04 (0.98–1.09)	0.123	958	83.5	1.08 (1.00–1.17)	0.045	1.21 (1.08–1.37)	0.001
≥65	423	87.1	1	-	1	-	170	77.1	1	-	1	-
*P*-trend				0.246		0.109				0.002		0.033
Sex												
Men	2,850	88.8	1	-	1	-	925	77.0	1	-	1	-
Women	2,987	87.7	0.98 (0.97–1.00)	0.095	0.97 (0.93–1.00)	0.100	2,676	86.6	1.12 (1.09–1.16)	<0.01	1.19 (1.13–1.26)	<0.001
Residence ^c^												
Urban	1,563	84.5	1	-	1	-	1,058	80.7	1	-	1	-
Rural	4,274	89.7	1.06 (1.03–1.09)	<0.001	1.05 (1.00–1.10)	0.033	2,543	85.2	1.05 (1.01–1.10)	0.014	1.04 (0.98–1.10)	0.165
Ethnicity ^d^												
Lao	3,482	89.0	1	-	1	-	2,255	85.6	1	-	1	-
Others	2,355	87.2	0.97 (0.95–1.00)	0.102	0.97 (0.94–1.00)	0.151	1,346	81.1	0.94 (0.91–0.98)	0.003	0.97 (0.92, 1.03)	0.457
Religion												
Buddhist	4,317	89.1	1	-	1	-	2,793	85.7	1	-	1	-
Others	1,364	84.8	0.95 (0.92–0.98)	0.001	0.95 (0.91–1.00)	0.562	728	76.4	0.89 (0.85–0.93)	<0.001	0.83 (0.74–0.93)	0.002
None^e^	155	100.0	-	-	-	-	79	100.0	-	-	-	-
Marital status												
Currently married	4,614	88.8	1	-	1	-	4,614	88.8	1	-	1	-
Others^f^	1,221	86.4	0.97 (0.94–0.99)	0.035	0.91 (0.86–0.96)	0.001	1,221	86.3	0.99 (0.96–1.02)	0.743	0.87 (0.79–0.96)	0.005
Educational level												
Never attended school	1,036	89.9	1.29 (1.13–1.49)	<0.001	1.28 (0.95–1.72)	0.099	1,036	89.8	1.34 (1.11–1.61)	0.002	1.72 (0.98–3.02	0.058
Primary school	2,422	88.9	1.28 (1.11–1.48)	<0.001	1.24 (0.94–1.65)	0.124	2,421	88.9	1.32 (1.09–1.60)	0.003	1.63 (0.95–2.78)<	0.070
Secondary school	1976	87.9	1.27 (1.10–1.46)	<0.001	1.25 (0.94–1.66)	0.116	1975	87.9	1.33 (1.10–1.61)	0.002	1.74 (1.01–3.01)	0.044
High school or higher	87	69.1		-	1	-	87	69.9	1	-	1	-
*P*-trend				<0.005		0.349				0.025		<0.001
Average income per household member per day in US dollars												
<1.9	1,396	89.0	1.04 (1.01–1.07)	0.004	1.03 (1.00–1.06)	0.024	1,363	88.9	1.06 (0.99–1.14)	0.079	1.09 (1.02–1.15)	0.003
≥1.9	911	85.0	1	-	1	-	819	85.1	1	-		

a Represents the weighted percentage.

b Model adjusted for age, sex, residence, ethnicity, religion, marital status, and educational level.

c “Rural” category for “Residence” including with and without roads.

d “Others” category for “Ethnicity” including PhouThai, Khermou, Khamu, Khmu, Leu, Mong, etc.

e “None” category for Religion was removed during the analysis due to 100% second-hand smoke exposure in this category.

f “Others” category for “Marital status” including single or never married, divorced or widowed or separated.

Second-hand smoke exposure at home was considered present if the participant’s responses to the question about having seen someone smoke inside the home was either “daily,” “weekly” or “monthly,” and was considered absent if the participant’s response was “less than monthly” or “no.” N represents the unweighted count (sample size).

**TABLE 2:** Prevalence of secondhand smoke exposure inside the workplace, Lao People’s Democratic Republic, 2015.

Characteristics	All participants (smokers and non-smokers)						Non-smokers					
	Unweighted count (N)	Weighted %^a^	Unadjusted prevalence ratio (95% CI)	*p-*Value	Adjusted prevalence ratio^b^ (95% CI)	*p-*Value	Unweighted count (N)	Weighted %^a^	Unadjusted prevalence ratio (95% CI)	*p-*Value	Adjusted prevalence ratio^b^ (95% CI)	*p-*Value
Total (N = 1,139)	714	63.0					396	54.0				
Age group (years)												
15–24	115	55.0	1	-	1	-	99	51.7	1	-	1	-
25–44	360	61.5	1.16 (1.01–1.34)	0.033	0.98 (0.88–1.08)	0.746	214	53.2	1.03 (0.86–1.23)	0.746	0.96 (0.81–1.13)	0.645
≥45	239	70.7	1.33 (1.15–1.53)	<0.001	1.00 (0.90–1.12)	0.864	83	59.5	1.15 (0.99–1.33)	0.067	0.93 (0.81–1.05)	0.270
*P*-trend				<0.001		0.805				0.072		0.650
Sex												
Men	441	65.5	1	-	1	-	159	50.6	1	-	1	-
Women	273	59.5	0.90 (0.82–0.99)	0.048	0.90 (0.83–0.97)	0.014	237	56.7	1.11 (0.97–1.28)	0.101	0.97 (0.84–1.11)	0.678
Residence^c^												
Urban	259	53.6	1	-	1	-	160	46.3	1	-	1	-
Rural	455	69.8	1.30 (1.13–1.49)	0.002	1.12 (0.97–1.29)	0.117	236	60.5	1.30 (1.07–1.58)	0.006	1.14 (0.93–1.41)	0.184
Ethnicity^d^												
Lao	548	63.7	1	-	1	-	316	54.8	1	-	1	-
Others	166	60.5	0.94 (0.84–1.06)	0.378	0.93 (0.83–1.04)	0.235	80	50.8	0.92 (0.78–1.09)	0.368	0.87 (0.72–1.06)	0.182
Religion												
Buddhist	616	62.3	1	-			350	53.5	1	-		
Others	42	53.6	1.10 (0.97–1.25)	0.118			25	49.2	1.10 (0.88–1.39)	0.385		
None^e^	56	89.2	-	-			21	78.3	-	-		
Marital status												
Currently married	547	65.8	1	-	1	-	263	55.7	1	-	1	-
Others^f^	165	55.4	0.84 (0.73–0.96)	0.011	0.95 (0.87–1.04)	0.332	132	49.2	0.91 (0.77–1.07)	0.260	0.96 (0.86–1.08)	0.583
Educational level ^g^												
Primary school or lower	293	79.6	1.38 (1.26–1.51)	<0.001	1.20 (1.09–1.32)	<0.002	137	71.5	1.38 (1.23–1.55)	<0.001	1.21 (1.05–1.40)	0.007
Secondary school or higher	318	57.4	1	-	1	-	206	51.5	1	-	1	-
*P*-trend				<0.001		0.002				<0.001		0.012
Average income per household member per day in US dollars												
<1.9	111	75.9	1.17 (1.02–1.34)	0.019			27	54.7	1.04 (0.77–1.40)	0.770		
≥1.9	146	64.5	1	-			25	52.3	1	-		
Occupation												
Unemployed & Non-farm self-employed	23	69.7	1.15 (0.87–1.53)	0.308	1.30 (1.15–1.48)	<0.001	8	59.3	1.24 (0.76–2.02)	0.376	0.89 (0.68–1.16)	0.412
Government sector	81	60.2	1	-	1	-	33	47.6	1	-	1	-
Non-government sector	39	69.3	1.15 (0.90–1.45)	0.241	0.97 (0.80–1.17)	0.768	14	53.1	1.11 (0.72–1.72)	0.624	1.07 (0.86–1.34)	0.502
Agriculture	110	79.7	1.32 (1.12–1.55)	<0.007	1.14 (0.98–1.33)	0.075	28	63.2	1.32 (0.92–1.90)	0.126	1.31 (1.08–1.59)	0.005

a Represents the weighted percentage.

b Model adjusted for age, sex, residence, ethnicity, marital status, and educational level.

c “Rural” category for “Residence” including with and without roads.

d “Others” category for “Ethnicity” including PhouThai, Khermou, Khamu, Khmu, Leu, Mong, etc.

e “None” category for Religion was removed during the analysis due to 100% second-hand smoke exposure in this category.

f “Others” category for “Marital status” including single or never married, divorced or widowed or separated.

g “Primary school or earlier” category for “Educational level” contains those who never attended the school and those going to primary school. “Secondary school or higher” category contains those going to secondary school, high school or higher.

Second-hand smoke exposure at workplaces was considered present if the participant’s response when asked about seeing someone smoke inside the building where they work during the past 30 days was “yes” and was considered absent if the participant’s response was “no”. CI, confidence interval. N represents the unweighted count (sample size)

Participants with lower education levels were more likely to report SHS exposure: 89.9% of those who never attended any school reported SHS exposure at home and 79.6% of those who completed primary school or lower reported SHS exposure at workplaces. Having an average income per household member per day below $1.9 US was associated with a higher prevalence of SHS exposure both at home and in the workplace both among all participants and among non-smokers.

In the adjusted analysis, being female [prevalence ratio (PR) = 1.19, 95% CI: 1.13–1.26], having a lower education level, and having an average income per household <$1.9 US (PR = 1.09, 95% CI: 1.02–1.15) remain associated with SHS exposure at home among non-smokers (Table 1). Also, in the adjusted analysis, completing primary school or a lower education level (PR = 1.21, 95% CI: 1.05–1.40), and having an agriculture occupation (PR = 1.31, 95% CI: 1.08–1.59) were associated with SHS exposure at their workplaces among non-smokers (Table 2); sex was not associated with SHS exposure at the workplace among non-smokers.

The weighted prevalence of SHS exposure at any indoor public place (including inside government offices, health care facilities, restaurants or food stores, and public transportation vehicles) was 99.2%, with the highest SHS exposure prevalence reported at restaurants or food stores (57.7%), followed by government offices (56.2%), public transportation vehicles (31.6%), and health care facilities (11.7%) (Table 3). Although men were more likely than women to report SHS exposure for each indoor public place category, the overall SHS exposure at any indoor public place was similarly high (>99%). Likewise, the overall SHS exposure at any indoor public place was comparably high for both rural and urban residents.

**TABLE 3:** Prevalence of secondhand smoke exposure at indoor public places, National Adult Tobacco Survey, Lao People’s Democratic Republic, 2015.

Characteristics	Any public place^a^ (N = 1965) %	Government buildings or offices (N = 1,162) %	Health care facilities (N = 128) %	Restaurants or food stores (N = 943) %	Public transportation vehicles (N = 156) %
Total	99.2	56.2	11.7	57.7	31.6
Age group					
15–24	99.4	40.7	8.5	64.0	28.7
25–44	99.6	56.1	13.4	59.8	36.7
45–64	98.4	62.2	11.0	50.0	27.3
≥65	100.0	69.0	10.1	43.5	30.0
*p* Value		<0.001	0.342	<0.001	0.272
Sex					
Men	99.1	60.8	13.9	62.1	33.3
Women	99.4	49.1	9.8	52.1	29.3
*p* Value		<0.001	0.013	<0.001	0.213
Residence ^b^					
Urban	99.3	45.3	11.0	57.2	24.9
Rural	99.1	63.0	12.0	58.1	35.1
*p* Value		<0.001	0.253	0.418	0.021
Ethnicity ^c^					
Lao	99.4	58.1	10.0	60.2	35.2
Others	98.9	54.9	14.1	52.2	27.0
*p* Value		0.198	0.001	0.001	0.022
Religion					
Buddhist	99.3	57.6	10.7	58.0	33.0
Others	99.0	54.3	14.8	57.4	27.2
None	100.0	58.7	12.7	40.1	-
*p* Value		0.250	0.012	0.136	0.073
Educational level					
Never attended school	99.5	73.1	11.9	48.6	18.8
Primary school	99.3	64.4	12.2	52.1	33.7
Secondary school	99.0	54.3	12.8	61.0	34.4
High school or higher	100.0	39.0	8.0	65.1	37.0
*p* Value		<0.001	0.412	<0.001	0.086

a Secondhand smoke exposure at any public place includes exposure at any of the four subsequent locations individually listed.

b “Rural” category for “Residence” including areas with and without roads.

c “Others” category for “Ethnicity” includes PhouThai, Khermou, Khamu, Khmu, Leu, Mong, etc.

N represents the unweighted count (sample size) with the weighted percentage.

## Discussion

The study’s main finding is that SHS exposure at home and in the workplace among all participants and non-smokers in Lao PDR is higher than exposures in other South-East Asian countries. Specifically, among all participants, the prevalence of SHS exposure at home in Lao PDR (88.3%) was higher than that in Bangladesh (54.9%), India (40.0%), The Philippines (54.4%), Thailand (33.2%), and Vietnam (73.1%) [Global Adult Tobacco survey (GATS) 2008–2010] [15]. Similarly, for non-smokers, SHS exposure at home in Lao PDR (83.9%) exceeds that in Bangladesh (48.4%), India (34.7%), The Philippines (44.8%), Thailand (25.3%), and Vietnam (67.6%) (GATS 2008–2010) [15]. While SHS exposure at workplaces among all participants in Lao PDR (63.0%) is similar to that in Bangladesh (62.2%), it is higher than that in India (29.9%), The Philippines (32.6%), Thailand (27.2%) (GATS 2008–2010) [15], and Vietnam (42.6%, NATS 2015) [16]. Similarly, among non-smokers SHS exposure at workplaces in Lao PDR (54.0%) is comparable to that in Bangladesh (54.6%), but higher than that in India (26.1%), The Philippines (28.0%), Thailand (23.6%) (GATS 2008–2010) [15], and Vietnam (36.8%) (NATS 2015) [16]. Finally, SHS exposure among all participants inside government buildings in Lao PDR (56.2%) is higher than in Bangladesh (43.3%), India (26.2%), The Philippines (25.5%), Thailand (13.0%), or Vietnam (38.7%) (GATS 2008–2010) [15]. These results highlight the need for comprehensive smoke-free policies at workplaces in Lao PDR, and for encouraging no smoking inside homes. Smoke-free policies for workplaces not only reduce SHS exposures at work but can also reduce SHS exposure at home; employment in a smoke-free workplace is associated with living in a smoke-free home [17]. Additionally, more robust smoking restrictions in the workplace are associated with lower daily cigarette consumption and a greater intention to quit [18].

From 2006–2008, several ministries of Lao PDR (e.g., Ministry of Education, Ministry of Health, and Ministry of Public Works and Transport) issued smoke-free policies for governmental premises [10, 12], including educational and healthcare facilities. Despite these policies, SHS exposure at public places remains very high based on data reported herein; stricter enforcement of smoke-free policies may be needed. In addition, the Lao government should introduce measures to encourage other public places, such as restaurants or food stores, to adopt either smoke-free environments or designated smoking areas.

The high prevalence of SHS exposure at home reported by non-smokers aged 15–24 (86.7%) is of particular concern. Adolescents and young adults who are frequently exposed to SHS at home may normalize smoking behavior and thus may be more likely to initiate smoking [19, 20]; starting smoking at an early age is associated with nicotine dependence and sustained tobacco use [21]. Even if SHS exposure does not lead to smoking in this age group, it still poses significant health risks; SHS exposure is a preventable risk factor for the development of cardiovascular disease through the development of endothelial dysfunction [22], lung and breast cancer [23], upper and lower respiratory tract infections, asthma [6], and COPD [7]. Additionally, SHS exposure is associated with mental health issues in non-smoking adolescents, including stress, major depressive disorder, generalized anxiety, and attention-deficit/hyperactivity disorder [24]. Interventions to reduce SHS exposure among adolescents and young adults in Lao PDR are needed to prevent future tobacco use and associated morbidity.

An earlier study showed that smoking prevalence is higher in Lao men than in Lao women [9, 25], thus it is understandable that the present study shows a higher prevalence of SHS exposure at home for women who did not smoke. Women and children worldwide are disproportionately affected by SHS exposure; 28% of SHS-related deaths occur among children and 47% among women [3]. SHS exposure increases the risk of several adverse health outcomes for both women and children. SHS exposure during pregnancy can cause low birth weight, fetal growth retardation, delayed immune development, reductions in all phases of infant sleep cycles, and also negatively affect the infant’s neurological development [26, 27]. Maternal SHS exposure is also associated with stillbirth, preterm birth, spontaneous abortion, and birth defects [27]. The gender-based disparity in SHS exposure is particularly prevalent at home rather than at the workplace. This disparity may be related to the awareness of the harms associated with SHS exposure, which is found to be low in low-and middle-income countries, and the patriarchal family structure in Asia that may hinder women’s confidence and ability to challenge men’s smoking behavior or ask them to smoke outdoors [28]. In addition, there is a lack of voluntary restrictions on smoking in households in some of the Asian countries including Lao PDR [29]. Thus, educational interventions to raise awareness and adopt smoke-free homes are necessary to protect the health of women and children and to reduce this gender-based health disparity. Smoke-free policies at public places seem to influence smoke-free home adoption [30]. Therefore, efforts to implement smoke-free environments at indoor public places in Lao PDR may further protect women both at these public places and at home. While NATS is comprised of a nationally representative sample it has certain limitations: all variables were collected via self-report, which might be subject to recall bias or social desirability bias. SHS exposure was not confirmed by biomarkers, such as serum cotinine levels or salivary cotinine levels that are available and were used in other Asian countries [31]. There might be a possibility of under-reporting of SHS exposure among women and children assessed by self-reporting in this study. There is a possibility of smokers misreporting SHS exposure to be absent despite being exposed to their smoke. Some stratified analyses (e.g., SHS exposure among smokers) could not be performed due to the small sample sizes of the subgroups.

### Conclusion

Findings from the 2015 NATS showed that the SHS exposure at home and workplace among all participants and non-smokers in Lao PDR is higher than those in other South-East Asian countries. There is a need for more comprehensive smoke-free policies at government-owned workplaces and facilities, stricter enforcement of these smoke-free policies, and strategies to encourage a smoke-free environment at homes and other public places such as restaurants or food stores. Educational interventions and campaigns are also needed to raise people’s awareness of the harms caused by SHS, which is necessary for the adoption of smoke-free homes or non-governmental environments to protect non-smokers’ health, particularly the health of women and children.
