# Supplementary material for: Secondhand Smoke Exposure in Lao People’s Democratic Republic: Results From the 2015 National Adult Tobacco Survey
Source: Int J Public Health. 2021 Dec 31;66:1604436. doi: 10.3389/ijph.2021.1604436 (PMC8758564; doi:10.3389/ijph.2021.1604436)
Supplement: Supplementary file 1 [file DataSheet1.docx]

**Supplementary Table 1:** Participants’ characteristics, NATS, 2015.

| Characteristics | All (Smokers and Non-smokers)  N (%) | Non-smokers  N (%) |
| --- | --- | --- |
| **Age group (years)** |  |  |
| 15-24 | 1399 (18.5) | 1230 (24.0) |
| 25-44 | 3235 (43.0) | 2282 (44.4) |
| 45-64 | 2378 (31.2) | 1354 (26.3) |
| ≥65 | 550 (7.3) | 271 (5.3) |
| **Sex** |  |  |
| Men | 3585 (47.4) | 1480 (29.0) |
| Women | 3977 (52.6) | 3657 (71.0) |
| **Residence** |  |  |
| Urban | 2370 (31.0) | 1764 (34.0) |
| Rural ^a^ | 5192 (69.0) | 3373 (66.0) |
| **Ethnicity** |  |  |
| Lao | 4542 (60.0) | 3194 (62.2) |
| Others ^b^ | 3020 (40.0) | 1943 (37.8) |
| **Religion** |  |  |
| Buddhist | 5602 (74.1) | 3921 (76.3) |
| Others | 1800 (23.8) | 1132 (22.1) |
| None ^c^ | 159 (2.1) | 83 (1.6) |
| **Marital status** |  |  |
| Currently married | 5862 (77.5) | 3731 (73.0) |
| Others ^d^ | 1697 (22.5) | 1404 (27.0) |
| **Educational level** |  |  |
| Never attended school | 1260 (17.7) | 837 (17.3) |
| Primary school | 3027 (42.6) | 1909 (39.5) |
| Secondary school | 2635 (37.0) | 1929 (40.0) |
| High school or higher | 192 (2.7) | 155 (3.2) |
| **Average income per household per day**  **In US dollars** |  |  |
| < 1.9 | 1702 (57.6) | 799 (54.9) |
| ≥ 1.9 | 1251 (42.4) | 657 (45.1) |
| **Occupation** |  |  |
| Unemployed and Non-farm self-employed | 1697 (24.1) | 1304 (27.6) |
| Government sector | 556 (8.0) | 380 (8.0) |
| Non-government sector | 1020 (14.5) | 658 (14.0) |
| Agriculture | 3755 (53.4) | 2374 (50.4) |

^a^ ‘Rural’ category for ‘Residence’ including with and without roads. ^b^ ‘Others’ category for ‘Ethnicity’ including PhouThai, Khermou, Khamu, Khmu, Leu, Mong, etc. ^c ‘^None’ category for Religion was removed during the analysis due to 100% second-hand smoke exposure in this category. ^d^ ‘Others’ category for ‘Marital status’ including single or never married, divorced or widowed or separated; N represents the unweighted count.

**Supplementary Table 2:** Prevalence of daily SHS exposure at home by demographic characteristics, NATS 2015.

| Characteristics | Daily SHS exposure at home among Smokers and Non-smokers | | Daily SHS exposure at home among Non-smokers | |
| --- | --- | --- | --- | --- |
|  | Unweighted N (%) | Weighted (%) | Unweighted N (%) | Weighted (%) |
|  | 4162 (63.0) | 63.0 | 2196 (51.2) | 51.5 |
| **Age group (years)** |  |  |  |  |
| 15-24 | 702 (60.0) | 60.0 | 559 (55.5) | 55.8 |
| 25-44 | 1705 (60.3) | 60.5 | 930 (48.6) | 48.7 |
| 45-64 | 1428 (67.3) | 67.5 | 600 (52.3) | 52.8 |
| ≥65 | 327 (67.6) | 66.6 | 107 (48.6) | 48.3 |
| **Sex** |  |  |  |  |
| Men | 2094 (65.4) | 65.5 | 412 (34.3) | 35.3 |
| Women | 2068 (60.9) | 60.8 | 1784 (57.9) | 57.8 |
| **Residence** |  |  |  |  |
| Urban | 988 (53.8) | 53.6 | 585 (45.0) | 45.0 |
| Rural ^a^ | 3174 (66.6) | 66.6 | 1611 (53.9) | 54.3 |
| **Ethnicity** |  |  |  |  |
| Lao | 2315 (59.5) | 59.3 | 1283 (49.0) | 49.0 |
| Others ^b^ | 1847 (68.2) | 68.7 | 913 (54.8) | 55.6 |
| **Religion** |  |  |  |  |
| Buddhist | 2905 (60.2) | 60.0 | 1616 (49.8) | 49.8 |
| Others | 1148 (71.0) | 72.0 | 541 (56.3) | 57.8 |
| None ^c^ | 108 (69.7) | 67.9 | 38 (48.1) | 46.0 |
| **Marital status** |  |  |  |  |
| Currently married | 3367 (65.0) | 65.0 | 1635 (51.9) | 52.1 |
| Others ^d^ | 793 (56.0) | 56.3 | 560 (49.4) | 49.8 |
| **Educational level** |  |  |  |  |
| Never attended school | 827 (71.5) | 72.6 | 441 (59.7) | 61.0 |
| Primary school | 1819 (66.9) | 66.6 | 891 (54.4) | 54.3 |
| Secondary school | 1281 (57.1) | 57.0 | 735 (46.6) | 46.8 |
| High school or higher | 54 (43.9) | 43.8 | 34 (34.7) | 34.6 |
| **Average income per household per day**  **In US dollars** |  |  |  |  |
| < 1.9 | 1054 (67.3) | 67.7 | 295 (42.7) | 43.6 |
| ≥ 1.9 | 621 (58.1) | 57.9 | 167 (32.2) | 33.3 |
| **Occupation** |  |  |  |  |
| Unemployed and Non-farm self-employed | 878 (62.7) | 63.0 | 569 (55.0) | 55.6 |
| Government sector | 219 (48.7) | 48.9 | 102 (35.0) | 35.4 |
| Non-government sector | 574 (64.5) | 64.0 | 285 (52.0) | 51.5 |
| Agriculture | 2248 (65.9) | 65.9 | 1079 (52.2) | 52.4 |

^a^ ‘Rural’ category for ‘Residence’ including with and without roads. ^b^ ‘Others’ category for ‘Ethnicity’ including PhouThai, Khermou, Khamu, Khmu, Leu, Mong, etc. ^c ‘^None’ category for Religion was removed during the analysis due to 100% second-hand smoke exposure in this category. ^d^ ‘Others’ category for ‘Marital status’ including single or never married, divorced or widowed or separated; N represents the unweighted count.
